# Semiresorbable biologic hybrid meshes for ventral abdominal hernia repair in potentially contaminated settings: lower risk of recurrence

**DOI:** 10.1007/s13304-022-01378-3

**Published:** 2022-10-12

**Authors:** Markus Goetz, Maria Jurczyk, Henrik Junger, Hans J. Schlitt, Stefan M. Brunner, Frank W. Brennfleck

**Affiliations:** Department of Surgery, University Medical Center Regensburg, Franz-Josef-Strauß-Allee 11, 93053 Regensburg, Germany

**Keywords:** Incisional hernia, Abdominal wall reconstruction, Biologic mesh, Wound contamination

## Abstract

In case of potential contamination, implantation of synthetic meshes in hernia and abdominal wall surgery is problematic due to a higher risk of mesh infection. As an alternative, a variety of different biologic meshes have been used. However, relevant data comparing outcome after implantation of these meshes are lacking. Between January 2012 and October 2021, biologic meshes were used for reconstruction of the abdominal wall in 71 patients with preoperative or intraoperative abdominal contamination. In this retrospective study, semiresorbable biologic hybrid meshes (BHM) and completely resorbable meshes (CRM) were compared and analyzed using a Castor EDC database. In 28 patients, semiresorbable biologic hybrid meshes were used; in 43 patients, completely resorbable meshes were used. Both groups showed no difference in age, gender, BMI, operation duration, hernia size and Charlson comorbidity index. The risk degree of surgical-site occurrences was graded according to the Ventral Hernia Working Group (VHWG) classification, and the median value was 3 (range 2–4) in the BHM group and 3 (range 2–4) in the CRM group. Hernia recurrence within 24 months after hernia repair was significantly lower in the BHM group (3.6% vs. 28.9%; *p* = 0.03), while postoperative complication rate, with respect to seromas in need of therapy (61.4% vs. 55.5%, *p* = 0.43) and operative revision (28.6% vs. 16.3%, *p* = 0.22) was not different in either group. Biologic hybrid meshes can be used safely in case of possible contamination. BHM seems to reduce the risk of hernia recurrence compared to completely resorbable biologic meshes, but this has to be investigated further.

## Introduction

In the surgical treatment of abdominal wall hernias, the use of meshes is recommended for larger hernias, and is associated with a significantly lower rate of hernia recurrence [1–3]. However, mesh infections represent a serious complication with a relevant necessity of reintervention and mesh removal, especially in contaminated wound situations [4, 5]. While synthetic meshes are commonly used, biologic meshes were invented as an alternative to reduce the risk of superinfection [6–9]. Since the advantage of biologic compared to synthetic meshes is discussed controversially in the literature [5, 10–15], clear guidelines on when to use biologic meshgrafts are lacking. Consequently, the decision about whether or not to use a biologic mesh, and which type depends on the intraoperative assessment of the individual surgeon. Many different types of biologic and biosynthetic meshes are available, most of them completely resorbable [6–8]. In our department, in the case of verified or possible wound contamination, we recently changed from using completely resorbable biologic meshes to OviTex^®^ 2s reinforced tissue meshes, consisting of a combination of ovine extracellular matrix and non-resorbable polypropylene fibers. The outcome in this group compared to all other patients with biologic meshgraft hernia repair was analyzed in this study.

## Methods

Between January 2012 and October 2021, 71 cases with biologic mesh implantation in the setting of preoperative or intraoperative possible wound contamination were identified. Only patients with reconstruction of the abdominal wall, which included ventral abdominal hernias and abdominal cavity closing after open abdomen situations, were enrolled in this study. Other indications for mesh implantation were excluded. To compare the outcome of the different meshes, the patients were retrospectively separated into a semiresorbable (BHM) and a completely resorbable meshes (CRM) group. The data were stored in a Castor EDC database and analyzed using IBM SPSS statistics 26. Statistical analysis was done using Student’s *t* test in case of normally distributed data, and otherwise the Mann–Whitney *U* test. The Chi-square test was used, if appropriate, to compare nominal variables. The recurrence-free survival rates were calculated with a Kaplan–Meier analysis and the log-rank method was used to compare these rates between groups. A *p* value of 0.05 or less was considered statistically significant. The study was approved by the ethics committee of the University of Regensburg, Germany (Nr. 19-1547-101).

## Materials

In total, four different types of biologic meshes were used. Three of them were completely resorbable and 1 semiresorbable:

- Resorbable
  o Strattice^™^ RTM: porcine-derived acellular dermal matrix
  o Permacol^™^: cross-linked, acellular porcine-derived dermal collagen matrix
  o SurgiMend^R^ integra: acellular collagen matrix derived from fetal and neonatal bovine dermis
- Semiresorbable
  o OviTex^™^ 2s: sterile bioscaffold composed of ovine-derived extracellular matrix and monofilament polypropylene.

VHWG classification of ventral hernias [4, 14, 15]:

- Grade 1 (low risk): low risk of complications, no history of wound infection
- Grade 2 (co-morbid): COPD, diabetes, immunosuppression, active smoker, obese
- Grade 3 (potentially infected): previous wound infection, stoma present, violation of the gastrointestinal tract
- Grade 4 (infected): infected mesh, septic dehiscence.

## Results

### Patient characteristics

Both groups showed no statistical difference in age, gender, BMI, Charlson Comorbidity Index, or hernia size, and only the presence of stomata was significantly higher in the CRM group (*p* = 0.01) (Table 1). The median age was 58 years (range 1–82 years) in the BHM group and 57 years (range 0–82 years) in the CRM group. 53.6% of the patients were male in the BHM cohort, and 60.5% in the CRM group. The median BMI was 28.8 kg/m^2^ (range 15–41.8 kg/m^2^) for patients treated with a biologic hybrid mesh compared to 27.5 kg/m^2^ (range 13.5–72.6 kg/m^2^) in the CRM group. Furthermore, a median of 3.5 points (range 0–8 points) were generated using the Charlson Comorbidity Index in the BHM group and 3 points (range 0–10 points) in the CRM group. While all patients had undergone abdominal surgical treatment in the past, the median number of previous operations was 4.5 (range 1–6) in the BHM group and 5 (range 1–6) in the CRM group. The risk degree of surgical-site occurrences according to the VHWG classification indicated a median value of 3 (range 2–4) in the BHM group and 3 (range 2–4) in the CRM group. The median hernia size was 220.7 cm^2^ (range 20.7–586.6 cm^2^) in the BHM group and 107.8 cm^2^ (range 0.2–505.5 cm^2^) in the CRM group. Preoperative wound conditioning, either through negative wound pressure therapy or regular lavage (Table 1), was performed in the BHM group and the CRM group in 42.9% and 37.2% of the cases, respectively (*p* = 0.64).

**Table 1:** Patient characteristics

	BHM group		CRM group		*P* value
Age, median (IQR), years	58 (1–82)		57 (0–82)		*0.84*
Gender	Male 15 (53.6%)	Female 13 (46.4%)	Male 26 (60.5%)	Female 17 (39.5%)	*0.57*
BMI, median (IQR), kg/m^2^	28.8 (15–41.8)		27.5 (13.5–72.6)		*0.71*
Charlson comorbidity index, median (IQR)	3.5 (0–8)		3 (0–10)		*0.6*
Pre-operation	Yes 28 (100%)	No 0 (0%)	Yes 43 (100%)	No 0 (0%)	
Number of pre-operations, median (IQR)	4.5 (1–6)		5 (1–6)		*0.54*
***Existing stoma***	Yes 2 (7.1%)	No 26 (92.9%)	Yes 14 (32.6%)	No 29 (67.4%)	0.01*
Type of hernia					
Primary	16 (57.1%)		28 (65.1%)		*0.73*
Recurrent	12 (42.9%)		15 (34.9%)		
Open abdomen	7 (25%)		11 (25.6%)		
Ventral hernia	21 (75%)		32 (74.4%)		
Hernia plane, median (IQR), cm^2^	220.7 (20.7–586.6)		107.8 (0.2–505.5)		*0.17*
Preoperative conditioning	Yes 12 (42.9%)	No 16 (57.1%)	Yes 16 (37.2%)	No 27 (62.8%)	*0.64*
VAC	12 (100%)		12 (75%)		
Lavage	0 (0%)		4 (25%)		
Antibiotics					
Preoperative	Yes 11 (39.3%)	No 17 (60.7%)	Yes 18 (42.9%)	No 24 (57.1%)	*0.82*
Postoperative	20 (71.4%)	8 (28.6%)	37 (88.1%)	5 (11.9%)	*0.08*

*Significant less patients in the BHM group had stomata (CI 0.95)

### Operative items

The median operation time was 162 min (range 51–493 min) in the BHM group and 157 min (range 37–511 min) in the CRM group. The mesh grafts were placed as intraperitoneal onlay mesh (IPOM), sublay, bridging, and onlay in 11 (39.3%), 9 (32.1%), 8 (28.6%), and 0 (0%) cases in the BHM group and in 26 (60.5%), 5 (11.6%), 11 (25.6%), and 1 (2.3%) cases in the CRM group, respectively (Table 2). The meshes were fixated with either a continuous resorbable suture at the fascial gap in case of interposition, or single stitches in the other positions. Complete fascial closure was achieved in 71.4% of the BHM-treated patients compared to 74.4% in the CRM group. In addition, the skin could primarily be closed in 26/28 (BHM group) and in 32/43 (CRM group) patients. Regarding drainage positioning, drains were placed subcutaneously and mesh-located in the BHM group in a significantly higher percentage of cases (88.2% vs. 58.1%, *p* = 0.00; 53.6% vs. 14%, *p* = 0.04), while both groups did not differ in intraabdominal position rate (Table 2).

**Table 2:** Operative parameters

	BHM group		CRM group		*P* value
Duration median (IQR), min	162 (51–493)		157 (36–511)		*0.55*
Mesh position					
IPOM	11 (39.3%)		26 (60.5%)		*0.35*
Sublay	9 (32.1%)		5 (11.6%)		
Bridging	8 (28.6%)		11 (25.6%)		
Onlay	0 (0%)		1 (2.3%)		
Fascial closure	Yes 20 (71.4%)	No 8 (28.6%)	Yes 32 (74.4%)	No 11 (25.6%)	*0.79*
Primary wound closure	Yes 26 (92.9%)	No 2 (7.1%)	Yes 32 (74.4%)	No 11 (25.6%)	*0.06*
Drainage					
Intraabdominal	Yes 16 (57.1%)	No 12 (42.9%)	Yes 27 (62.8%)	No 16 (37.2%)	*0.64*
Subcutaneous	23 (82.1%)	5 (17.9%)	25 (58.1%)	18 (41.9)	0.00*
Mesh	15 (53.6%)	13 (46.4%)	6 (14%)	37 (86%)	0.04**
Sterile operation site	Yes 20 (71.4%)	No 8 (28.6%)	Yes 23 (54.8%)	No 19 (45.2%)	*0.17*

*In significant more patients subcutaneous drainage were placed in the BHM group (CI 0.95)

**In significant more patients mesh-located drainage were placed in the BHM group (CI 0.95)

### Postoperative course

**Fig. 1:**
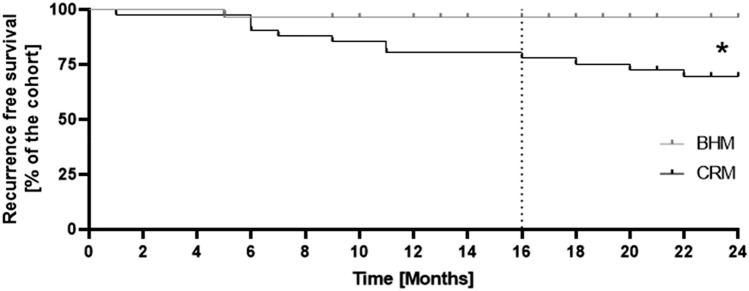
Shown is a Kaplan–Meier curve of recurrence-free survival within the first 24 months after hernia repair. Hernia recurrence is significantly lower (*p* = 0.03) in the BHM group (median follow-up in the BHM group is 16 months; marked with dotted line)

The median hospital stay was 28 days (range 6–271 days) for all patients with 29.5 days (range 6–136 days) in the BHM group and 27 days (range 7–271 days) in the CRM group (*p* = 0.35). With 46.4% vs. 20.9% a higher number of patients developed a seroma, located either subcutaneously or around the mesh in the BHM group. However, statistical significance was just missed (*p* = 0.08). Despite this, no difference in the necessity of interventional treatment (BHM 61.4% vs. CRM 55.6%) or the rate of positive microbiological findings in the seromas (BHM 15.4% vs. CRM 15.1%) was seen when comparing both groups. Operative revisions had to be performed in 8 (28.6%) cases in the BHM group and 7 (16.3%) cases in the CRM group (*p* = 0.22). The median follow-up was 16 months (range 9–28 months) in the BHM group and 31.5 months (range 5–125 months) in the CRM group with a total hernia recurrence rate of 7.1% in the BHM group and 32.6% in the CRM group, respectively. Due to the different length of the follow-up, hernia recurrence within 24 months after hernia repair was chosen for statistical analysis for better comparability. During this period, hernia recurrence was significantly lower in the BHM group, with specifically only 1 patient (3.6%) in the BHM group and 12 patients (28.9%) in the CRM group (Table 3; *p* = 0.03). A detailed stratification of hernia recurrence considering the respective operation technique is shown in Table 4. The median time to diagnosis of recurrence was 5 months in the BHM group vs. 10 months (range 1–22 months) in the CRM group (Fig. 1). At the data lock point, 14.3% of patients in the BHM group and 7% in the CRM group, respectively, still reported discrete abdominal pain, with a median VAS score of 4 (range 3–6) in the BHM group and 4 (range 3–6) in the CRM group (*p* = 1.0).

**Table 3:** Postoperative parameters

Value	BHM group					CRM group					*P* value
Postoperative seroma	Yes 13 (46.4%)		No 15 (53.6%)			Yes 9 (20.9%)		No 34 (79.1%)			*0.08*
Seroma localisation											
Subcutaneous	9 (69.2%)					5 (55.6%)					*0.54*
Under the mesh	4 (30.8%)					4 (44.4%)					
Seroma therapy											
None	5 (38.5%)					4 (44.4%)					*0.43*
Drainage	4 (30.8%)					5 (55.6%)					
Operative	4 (30.8%)					0 (0%)					
Operative revision	8 (28.6%)		20 (71.4%)			7 (16.3%)		36 (83.7%)			*0.22*
Hernia recurrence after 24 month follow-up	1 (3.6%)		27 (96.4%)			12 (28.9%)		31 (72.1%)			0.03*
Duration till recurrence, median (IQR), months	5					10 (1–22)					*0.39*
Actual pain	Yes 4 (14.3%)		No 21 (75%)		Unknown 3 (10.7%)	Yes 3 (7%)		No 33 (76.7%)		Unknown 7 (16.3%)	*0.21*
VAS-Score	4 (3–6)					4 (3–6)					*1.0*
Ability to work	Yes 12 (42.9%)		No 10 (35.7%)		Unknown 6 (21.4%)	Yes 19 (44.2%)		No 16 (37.2%)		Unknown 8 (18.6%)	*0.83*

*Hernia recurrence rate after 24 months is significant lower in the BHM group (CI 0.95)

**Table 4:** Recurrence stratification considering OP-techniques

	BHM group Total number (hernia recurrence)	CRM group Total number (hernia recurrence)
Onlay	0	1 (1)
IPOM	11 (1)	26 (9)
Sublay	9 (0)	5 (1)
Bridging	8 (1)	11 (3)

### Laboratory results

**Fig. 2:**
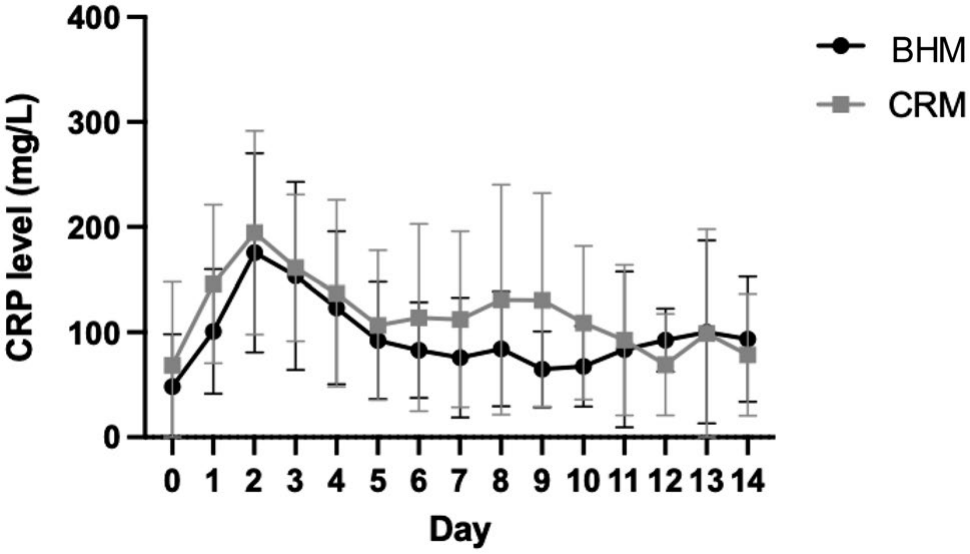
Both groups show a notable increase in the CRP score within the first 3 days after mesh implantation. Although slowly decreasing afterwards, CRP levels stay elevated over the next several days. No significant difference is seen between groups

**Fig. 3:**
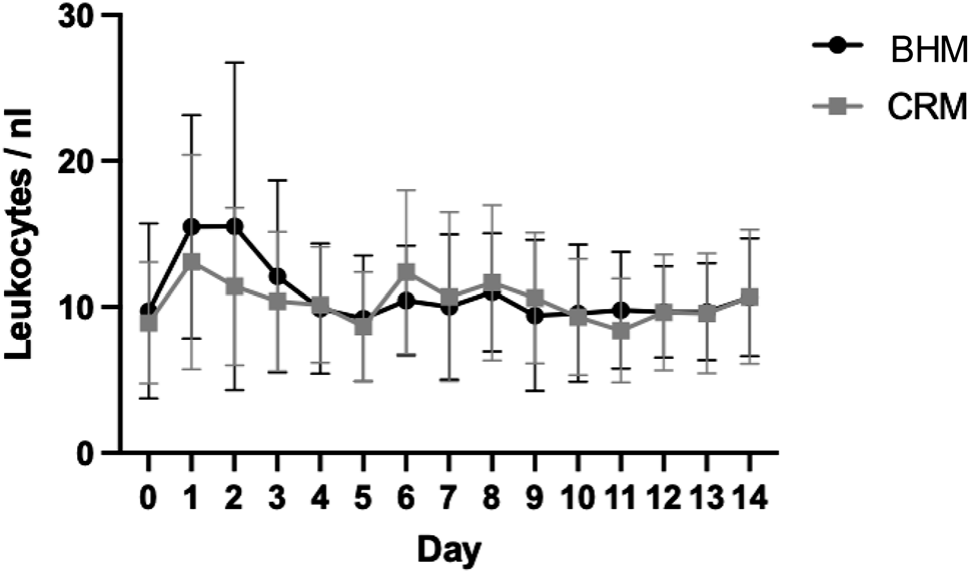
Similar to the CRP levels, the white blood cell count also increases until day 3 after the operation, and decreases over the following days. No statistical difference is seen between groups

**Fig. 4:**
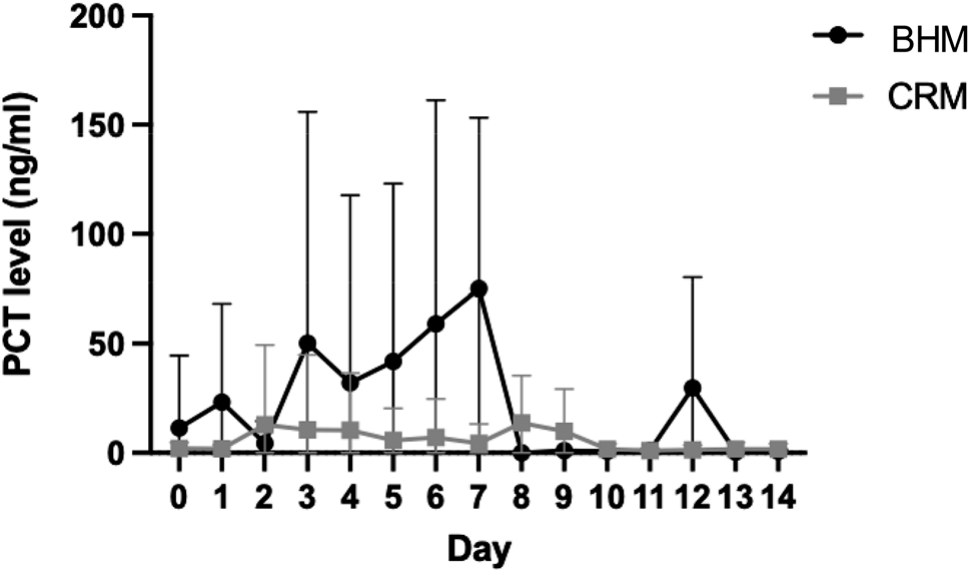
Shown is the PCT level course over the first 14 days. In the CRM group, the PCT level is higher than in the BHM group until postoperative day 10. Afterwards, the curves begin to align. No significant difference is reached

In both groups, a notable increase in CRP scores (Fig. 2), white blood cell counts (Fig. 3), and procalcitonin (PCT) (Fig. 4) was seen over the initial 2–3 days after mesh implantation, with no significant differences between the BHM and the CRM group. In the further postoperative course, all three parameters steadily decreased in both groups, but never reached the normal range over the first 14 days.

## Discussion

The results of this study suggest that semiresorbable biologic hybrid meshes show a significantly lower hernia recurrence rate (*p* = 0.03) in a mid-term follow-up period when compared to completely resorbable biologic meshgrafts in potentially contaminated wounds. Comparable results regarding hernia recurrence rates for meshes with non-resorbable components are found in the current literature [5, 9–13, 16, 17]. Although most studies only compare non-resorbable synthetic meshes with different completely resorbable biologic meshes [5, 10–13], Parker et al. [9] and Timmer et al. [16] as well as the 12 months analysis of the ongoing prospective BRAVO study [17] present low rates of hernia recurrence and surgical-site occurences requiring intervention for biologic hybrid meshes. Confirming the low rate of surgical-site complications in these studies, both groups in our study show a comparable safe use in complex patients and potentially contaminated wound situations. We observed a slightly higher rate of operative revisions without statistical significance in the BHM group (28.6% vs 16.3%), mostly due to wound infections, which is an acceptable level for such complex patients when considering the bigger defect size (220.7 cm^2^ vs. 107.8 cm^2^) and is consistent with the current literature analyzing biologic meshgrafts [5–9]. Moreover, mesh removal was not necessary in either the BHM or CRM group. A further complication seen twice as much without reaching the significance level in the BHM group in our study is the development of postoperative seromas. This is not necessarily related to contamination, but could also be due to an early immunological reaction, which is triggered more intensively by the monofilament polypropylene fibers, and was previously observed in a non-human model for these meshes [18]. This assumption is supported by the low rate of microbiological findings after drainage of these seromas and the similar need of reintervention in both groups. Therefore, the clinical condition of the patient and signs of a systemic infection in combination with raised infection parameters should be evaluated before considering an intervention.

There are, of course, some limitations to our study mainly because of the retrospective study design without randomization, which must be considered. Generally, a possible bias in patient selection cannot be completely excluded, although the presence of stomata was the only significant difference in patient characteristics between both groups. Furthermore, an increasing experience of the surgeons due to non-contemporaneous mesh usage is possible and the patient number (28 vs. 43) as well as the follow-up period (16 months vs. 31.5 months) differs in both groups.

Nevertheless, a clear benefit of this study is the comparison of a new semiresorbable biologic hybrid mesh with other kinds of completely resorbable biologic meshes, which is a valuable comparison that has not yet been done before. However, since only ovine biologic hybrid meshes were used in this study, all conclusions must be limited to this type of meshgraft.

## Conclusion

In summary, we suggest that semiresorbable biologic hybrid meshes are safe to use in complex patients with possible wound contamination and potentially reduce the risk of hernia recurrence compared to CRM in a mid-term follow-up period. Despite the heterogeneity of the presented retrospective study, our data show actual clinical courses of complex patients. However, for definitive clinical and economic conclusions, further prospective randomized trials and long-term follow-up data are required.

## Abbreviations

BHM: Semiresorbable biologic hybrid meshes
CRM: Completely resorbable meshes
VHWG: Ventral hernia working group
BMI: Body mass index
%: Percent
kg/m^2^: Kilogram per square meters
cm^2^: Square centimeters
min: Minutes
IPOM: Intraperitoneal onlay mesh
vs.: Versus
CRP: C-reactive protein
PCT: Procalcitonin
VAS: Visual analogue scale
fig: Figure
